# Supplementary material for: Restoration of Sestrin 3 Expression Mitigates Cardiac Oxidative Damage in Ischemia–Reperfusion Injury Model
Source: Antioxidants (Basel). 2025 Jan 7;14(1):61. doi: 10.3390/antiox14010061 (PMC11763094; doi:10.3390/antiox14010061)

Supplementary Table S1. Predicted miR-25 target genes and their characteristics

Predicted miR-25 Target Genes and Their Characteristics

Description	geneID
craniofacial suture morphogenesis	FOXN3/INSIG1/RAB23/MMP16
rhythmic process	AXL/PLEKHA1/FBXW7/PER2/MYCBP2/ADRB1/KAT2B/ZFHX3/PTEN/ROBO2/KCND2
bone development	FOXN3/INSIG1/MEF2D/RAB23/FBXW7/BMPR2/FBN1/PTGER4/MMP16
positive regulation of catabolic process	BCL2L11/FBXW7/TOB1/SOCS5/CLEC16A/EDEM1/NEDD4L/PRKCE/SESN3/PTEN/WWP2/CPEB3/FMR1
potassium ion transmembrane transport	SLC9A2/KCNA1/SLC12A5/DPP10/NEDD4L/SLC12A2/PTEN/KCND2/WWP2
skeletal system morphogenesis	PLEKHA1/FOXN3/INSIG1/MEF2D/RAB23/HOXD10/SATB2/BMPR2/MMP16
regulation of cation transmembrane transport	JPH2/ATP2B4/KCNA1/MYO5A/DPP10/NEDD4L/PRKCE/UBASH3B/PTEN/WWP2/FMR1
appendage development	BCL2L11/ITGA6/ASPH/HOXD10/SOX4/AFF3/BMPR2/KAT2B
limb development	BCL2L11/ITGA6/ASPH/HOXD10/SOX4/AFF3/BMPR2/KAT2B
regulation of cytoplasmic translation	CPEB2/CPEB4/CPEB3/FMR1
bone morphogenesis	FOXN3/INSIG1/MEF2D/RAB23/BMPR2/MMP16
regulation of ion transmembrane transport	JPH2/ATP2B4/KCNA1/MYO5A/PER2/DPP10/NEDD4L/PRKCE/UBASH3B/PTEN/KCND2/WWP2/FMR1
potassium ion transport	SLC9A2/KCNA1/SLC12A5/DPP10/NEDD4L/SLC12A2/PTEN/KCND2/WWP2
filopodium assembly	ITGA6/WASL/GPM6A/PPP1R9A/FMR1
dendrite development	MARK1/SLC12A5/WASL/NEDD4L/PTPRD/PTEN/PPP1R9A/CPEB3/FMR1
dendritic spine development	SLC12A5/WASL/PTEN/PPP1R9A/CPEB3/FMR1
calcium ion transmembrane transport	JPH2/ATP2B4/MYO5A/ATP2A2/ITGAV/ITPR1/PRKCE/UBASH3B/GPM6A/FMR1
locomotory behavior	NOVA1/ARRDC3/HOXD10/MYO5A/PRKCE/ZFHX3/PTEN/KCND2
synaptic vesicle cycle	FCHO2/ERC2/SLC17A6/BSN/ATP2A2/PTEN/SYT1/FMR1
spontaneous synaptic transmission	SLC12A2/PPP1R9A/SYT1
actin filament-based movement	DSC2/ACTC1/MYO1B/MYO5A/WASL/ATP2A2/NEDD4L
multicellular organismal signaling	DSC2/ATP2B4/ASPH/KCNA1/ATP2A2/ITPR1/KCND2/FMR1
maintenance of location	JPH2/INSIG1/FBXW7/MYO5A/SLC30A7/ITGAV/FBN1/ITPR1/PRKCE/UBASH3B
regulation of dendrite development	MARK1/NEDD4L/PTPRD/PTEN/PPP1R9A/CPEB3/FMR1
locomotor rhythm	ZFHX3/PTEN/KCND2
post-embryonic animal organ development	BCL2L11/KLF4/FBN1
positive regulation of cell projection organization	ITGA6/WASL/BMPR2/NEDD4L/PTPRD/GPM6A/PPP1R9A/ROBO2/SYT1/CPEB3/FMR1
regulation of protein ubiquitination	ARRDC3/FBXW7/PER2/SOX4/MYCBP2/PRKCE/PTEN/CUL3
acidic amino acid transport	PER2/SLC17A6/SLC6A1/SLC12A2/SYT1
regulation of metal ion transport	JPH2/ATP2B4/KCNA1/MYO5A/DPP10/NEDD4L/PRKCE/UBASH3B/PTEN/WWP2/FMR1
cellular response to peptide	BCL2L11/CPEB2/INSIG1/KLF4/APPL1/MYO5A/FBN1/KAT2B/SESN3/PTEN/RPS6KB1
vesicle-mediated transport in synapse	FCHO2/ERC2/SLC17A6/BSN/ATP2A2/PTEN/SYT1/FMR1
calcium-mediated signaling	JPH2/ATP2B4/MYO5A/PTPRJ/ATP2A2/ITPR1/PPP1R9A/DYRK2
cellular response to insulin stimulus	CPEB2/INSIG1/APPL1/MYO5A/KAT2B/SESN3/PTEN/RPS6KB1
heart contraction	DSC2/ACTC1/ATP2B4/ASPH/ATP2A2/ADRB1/ITPR1/NEDD4L/KCND2
neurotransmitter transport	ERC2/PER2/SLC17A6/SLC6A1/ATP2A2/PPP1R9A/SYT1/FMR1
positive regulation of neuron projection development	ITGA6/BMPR2/NEDD4L/PTPRD/PPP1R9A/ROBO2/SYT1/CPEB3/FMR1
vesicle transport along actin filament	MYO1B/MYO5A/WASL
sodium ion import across plasma membrane	SLC9A2/SLC6A1/SLC12A2
regulation of filopodium assembly	WASL/GPM6A/PPP1R9A/FMR1
cytosolic calcium ion transport	JPH2/ATP2B4/MYO5A/ATP2A2/ITPR1/PRKCE/UBASH3B
heart process	DSC2/ACTC1/ATP2B4/ASPH/ATP2A2/ADRB1/ITPR1/NEDD4L/KCND2
synapse organization	ERC2/MYO5A/SLC6A1/WASL/BSN/PTPRD/PTEN/GPM6A/PPP1R9A/ROBO2/CTTNBP2
actin filament organization	ACTC1/CD2AP/MYO1B/MYO5A/WASL/FMN2/PRKCE/PTGER4/PPP1R9A/CUL3/IQGAP2
regulation of blood circulation	DSC2/ATP2B4/ASPH/PER2/ATP2A2/ADRB1/BMPR2/ITPR1/KCND2
post-embryonic development	PLEKHA1/BCL2L11/KLF4/FBN1/ITPR1
regulation of protein modification by small protein conjugation or removal	ARRDC3/FBXW7/PER2/SOX4/MYCBP2/PRKCE/PTEN/CUL3
phosphatidylinositol metabolic process	PLEKHA1/RAB14/MTMR9/PIKFYVE/SOCS6/SOCS5/PTEN
circadian behavior	ADRB1/ZFHX3/PTEN/KCND2
actin filament-based transport	MYO1B/MYO5A/WASL
regulation of membrane potential	DSC2/KCNA1/SLC25A36/ATP2A2/ADRB1/NEDD4L/PTEN/PPP1R9A/KCND2/WWP2/FMR1
negative regulation of transporter activity	NEDD4L/PRKCE/PTEN/WWP2/FMR1

Predicted miR-25 Target Genes and Their Characteristics

Description	geneID
rhythmic behavior	ADRB1/ZFHX3/PTEN/KCND2
cell cycle G1/S phase transition	ATP2B4/KLF4/APPL1/FBXW7/SOX4/PTEN/RPS6KB1/CUL3/RBL2
ER-nucleus signaling pathway	BCL2L11/INSIG1/FBXW7/ATP2A2
positive regulation of neuron differentiation	ITGA6/BMPR2/NEDD4L/PTPRD/PTEN/PPP1R9A/ROBO2/SYT1/CPEB3/FMR1
action potential	DSC2/KCNA1/ATP2A2/NEDD4L/KCND2/FMR1
regulation of potassium ion transmembrane transport	KCNA1/DPP10/NEDD4L/PTEN/WWP2
central nervous system neuron differentiation	HOXD10/NFIB/SOX4/MYCBP2/SATB2/PTEN/ROBO2
second-messenger-mediated signaling	JPH2/ATP2B4/ARRDC3/MYO5A/PTPRJ/ATP2A2/ADRB1/ITPR1/PTGER4/PPP1R9A/DYRK2
calcium ion transmembrane import into cytosol	JPH2/ATP2B4/MYO5A/ITPR1/PRKCE/UBASH3B
regulation of BMP signaling pathway	TRIM33/NEO1/TOB1/BMPR2/FBN1
positive regulation of cellular catabolic process	BCL2L11/FBXW7/TOB1/SOCS5/CLEC16A/EDEM1/SESN3/PTEN/CPEB3/FMR1
negative regulation of immune system process	AXL/APPL1/FBXW7/PTPRJ/WASL/DUSP10/SOCS6/SOCS5/FBN1/UBASH3B/PTGER4
cellular response to external stimulus	AXL/DSC2/ITGA6/MAP2K4/BMPR2/CLEC16A/SESN3/CPEB4/PTGER4
extracellular matrix organization	ADAMTSL3/NPNT/ITGA6/IBSP/ITGAV/ADAMTSL1/FBN1/COL19A1/MMP16/COL5A1
extracellular structure organization	ADAMTSL3/NPNT/ITGA6/IBSP/ITGAV/ADAMTSL1/FBN1/COL19A1/MMP16/COL5A1
inorganic cation import across plasma membrane	SLC9A2/ATP2B4/SLC12A5/SLC6A1/SLC12A2
inorganic ion import across plasma membrane	SLC9A2/ATP2B4/SLC12A5/SLC6A1/SLC12A2
negative regulation of locomotion	ATP2B4/KLF4/ARRDC3/PTPRJ/WASL/DUSP10/PTEN/PTGER4/SEMA6D/ROBO2
regulation of protein catabolic process	FBXW7/MYCBP2/SOCS5/CLEC16A/EDEM1/FMN2/NEDD4L/PTEN/WWP2/FMR1
divalent metal ion transport	JPH2/ATP2B4/MYO5A/SLC30A7/ATP2A2/ITGAV/ITPR1/PRKCE/UBASH3B/GPM6A/FMR1
cellular response to peptide hormone stimulus	CPEB2/INSIG1/APPL1/MYO5A/FBN1/KAT2B/SESN3/PTEN/RPS6KB1
regulation of ion transmembrane transporter activity	JPH2/KCNA1/MYO5A/NEDD4L/PRKCE/PTEN/WWP2/FMR1
cardiac conduction	DSC2/ATP2B4/ASPH/ATP2A2/ITPR1/KCND2
regulation of cell cycle G1/S phase transition	ATP2B4/KLF4/APPL1/FBXW7/SOX4/PTEN/RBL2
positive regulation of endocytosis	AXL/APPL1/WASL/NEDD4L/FMR1
regulation of calcium-mediated signaling	JPH2/ATP2B4/MYO5A/ITPR1/DYRK2
divalent inorganic cation transport	JPH2/ATP2B4/MYO5A/SLC30A7/ATP2A2/ITGAV/ITPR1/PRKCE/UBASH3B/GPM6A/FMR1
proteasomal protein catabolic process	CD2AP/FBXW7/HERC2/SOCS6/SOCS5/CLEC16A/EDEM1/NEDD4L/CUL3/WWP2/FMR1
negative regulation of transport	INSIG1/RAB23/APPL1/ITGAV/NEDD4L/PRKCE/PTEN/PTGER4/PPP1R9A/WWP2/FMR1
regulation of the force of heart contraction	ATP2B4/ATP2A2/ADRB1
peptidyl-tyrosine dephosphorylation	PTPRJ/DUSP10/PTPRD/UBASH3B/PTEN
positive regulation of neurogenesis	ITGA6/BMPR2/MAN2A1/NEDD4L/PTPRD/PTEN/PPP1R9A/ROBO2/SYT1/CPEB3/FMR1
regulation of transmembrane transporter activity	JPH2/KCNA1/MYO5A/NEDD4L/PRKCE/PTEN/WWP2/FMR1
startle response	KCNA1/PTEN/CSMD1
adenylate cyclase-activating adrenergic receptor signaling pathway	ATP2B4/ARRDC3/ADRB1
import across plasma membrane	SLC9A2/ATP2B4/PER2/SLC12A5/SLC6A1/SLC12A2
maintenance of location in cell	JPH2/INSIG1/MYO5A/SLC30A7/ITPR1/PRKCE/UBASH3B
calcium ion transport into cytosol	JPH2/ATP2B4/MYO5A/ITPR1/PRKCE/UBASH3B
regulation of potassium ion transport	KCNA1/DPP10/NEDD4L/PTEN/WWP2
neuromuscular process	HOXD10/KCNA1/MYCBP2/PTEN/CSMD1
circadian rhythm	FBXW7/PER2/MYCBP2/ADRB1/ZFHX3/PTEN/KCND2
negative regulation of osteoclast differentiation	FBXW7/FBN1/UBASH3B
response to insulin	CPEB2/INSIG1/APPL1/MYO5A/KAT2B/SESN3/PTEN/RPS6KB1
negative regulation of protein kinase B signaling	PLEKHA1/KLF4/PTPRJ/PTEN
axon guidance	CNTN4/PALLD/NEO1/NFIB/MYCBP2/BMPR2/SEMA6D/ROBO2
neuron projection guidance	CNTN4/PALLD/NEO1/NFIB/MYCBP2/BMPR2/SEMA6D/ROBO2
calcium ion transport	JPH2/ATP2B4/MYO5A/ATP2A2/ITGAV/ITPR1/PRKCE/UBASH3B/GPM6A/FMR1
positive regulation of filopodium assembly	WASL/GPM6A/FMR1
positive regulation of cell adhesion	NPNT/ITGA6/IBSP/PTPRJ/SOX4/DUSP10/ITGAV/SOCS5/PRKCE/ZFHX3
regulation of potassium ion transmembrane transporter activity	KCNA1/NEDD4L/PTEN/WWP2
positive regulation of protein catabolic process	FBXW7/SOCS5/EDEM1/NEDD4L/PTEN/WWP2/FMR1
negative regulation of response to external stimulus	KLF4/MFHAS1/DUSP10/SOCS5/SLC12A2/UBASH3B/PTEN/PTGER4/SEMA6D/ROBO2
regulation of transporter activity	JPH2/KCNA1/MYO5A/NEDD4L/PRKCE/PTEN/WWP2/FMR1

Predicted miR-25 Target Genes and Their Characteristics

Description	geneID
long-term synaptic depression	PTEN/PPP1R9A/FMR1
regulation of lipid kinase activity	KLF4/SOCS6/SOCS5/RBL2
regulation of synaptic vesicle cycle	BSN/ATP2A2/PTEN/SYT1/FMR1
cranial skeletal system development	FOXN3/INSIG1/RAB23/MMP16
negative regulation of calcium-mediated signaling	ATP2B4/ITPR1/DYRK2
adrenergic receptor signaling pathway	ATP2B4/ARRDC3/ADRB1
import into cell	SLC9A2/ATP2B4/PER2/SLC12A5/SLC6A1/SLC12A2/RPS6KB1
protein monoubiquitination	KBTBD8/PCGF3/NEDD4L/CUL3
cellular response to amino acid stimulus	IPO5/SESN3/CPEB4/CPEB3
negative regulation of cyclin-dependent protein serine/threonine kinase activity	IPO5/KAT2B/PTEN
phosphatidylinositol biosynthetic process	PLEKHA1/RAB14/MTMR9/PIKFYVE/PTEN
release of sequestered calcium ion into cytosol	JPH2/MYO5A/ITPR1/PRKCE/UBASH3B
regulation of neurotransmitter levels	ERC2/PER2/SLC6A1/ATP2A2/PPP1R9A/SYT1/FMR1
sodium ion transmembrane transport	SLC9A2/ATP2B4/SLC6A1/NEDD4L/PRKCE/SLC12A2
negative regulation of sequestering of calcium ion	JPH2/MYO5A/ITPR1/PRKCE/UBASH3B
neuronal action potential	KCNA1/KCND2/FMR1
negative regulation of cyclin-dependent protein kinase activity	IPO5/KAT2B/PTEN
regulation of translation	KBTBD8/CPEB2/PER2/SOX4/TOB1/QKI/CPEB4/RPS6KB1/CPEB3/FMR1
regulation of neurotransmitter transport	PER2/ATP2A2/PPP1R9A/SYT1/FMR1
positive regulation of protein ubiquitination	ARRDC3/FBXW7/MYCBP2/PTEN/CUL3
regulation of sequestering of calcium ion	JPH2/MYO5A/ITPR1/PRKCE/UBASH3B
regulation of cardiac conduction	ATP2B4/ASPH/ATP2A2/ITPR1
regulation of synapse structure or activity	SLC17A6/PTPRD/PTEN/GPM6A/PPP1R9A/ROBO2/CTTNBP2
modulation of chemical synaptic transmission	CNTN4/SLC6A1/ATP2A2/PRKCE/SLC12A2/PTEN/PPP1R9A/SYT1/CPEB3/FMR1
regulation of trans-synaptic signaling	CNTN4/SLC6A1/ATP2A2/PRKCE/SLC12A2/PTEN/PPP1R9A/SYT1/CPEB3/FMR1
regulation of synaptic transmission, GABAergic	SLC6A1/PRKCE/PTEN
positive regulation of ubiquitin-protein transferase activity	ARRDC3/FBXW7/PTEN
negative regulation of animal organ morphogenesis	FBXW7/NFIB/BMPR2
sodium ion transport	SLC9A2/ATP2B4/SLC17A6/SLC6A1/NEDD4L/PRKCE/SLC12A2
regulation of cell morphogenesis involved in differentiation	MYCBP2/BMPR2/NEDD4L/PTPRD/PTEN/SEMA6D/PPP1R9A/ROBO2
regulation of cellular component size	SLC12A5/WASL/BMPR2/PRKCE/SLC12A2/PTEN/SEMA6D/PPP1R9A/IQGAP2
mitral valve morphogenesis	SOX4/BMPR2
presynaptic membrane assembly	PTPRD/PTEN
sequestering of calcium ion	JPH2/MYO5A/ITPR1/PRKCE/UBASH3B
negative regulation of synaptic transmission	SLC6A1/PTEN/PPP1R9A/FMR1
positive regulation of dendrite development	PTPRD/PPP1R9A/CPEB3/FMR1
eye development	ATP2B4/KLF4/PDS5B/SLC17A6/BMPR2/FBN1/MAN2A1/GPM6A/COL5A1
central nervous system neuron axonogenesis	NFIB/MYCBP2/PTEN
regulation of amino acid transport	PER2/SLC6A1/SLC12A2
cellular response to acid chemical	IPO5/SESN3/CPEB4/CPEB3
negative regulation of protein kinase activity	IPO5/PTPRJ/DUSP10/SOCS5/KAT2B/UBASH3B/PTEN
regulation of response to wounding	KLF4/DUSP10/PRKCE/SLC12A2/UBASH3B/PTEN
visual system development	ATP2B4/KLF4/PDS5B/SLC17A6/BMPR2/FBN1/MAN2A1/GPM6A/COL5A1
synapse assembly	BSN/PTPRD/PTEN/GPM6A/PPP1R9A/ROBO2
negative regulation of ion transmembrane transporter activity	NEDD4L/PRKCE/PTEN/FMR1
positive regulation of proteolysis involved in cellular protein catabolic process	FBXW7/SOCS5/EDEM1/PTEN/FMR1
muscle system process	DSC2/NPNT/ACTC1/ATP2B4/KLF4/MAP2K4/ASPH/KCNA1/ATP2A2/NEDD4L
regulation of dendritic spine development	PTEN/PPP1R9A/CPEB3/FMR1
negative regulation of wound healing	DUSP10/SLC12A2/UBASH3B/PTEN
positive regulation of amine transport	SLC6A1/SLC12A2/SYT1
sensory system development	ATP2B4/KLF4/PDS5B/SLC17A6/BMPR2/FBN1/MAN2A1/GPM6A/COL5A1
cytoplasmic translational elongation	CPEB2/CPEB3
mitral valve development	SOX4/BMPR2

Predicted miR-25 Target Genes and Their Characteristics

Description	geneID
regulation of Fc receptor mediated stimulatory signaling pathway	APPL1/PTPRJ
presynaptic membrane organization	PTPRD/PTEN
negative regulation of adenylate cyclase-activating G protein-coupled receptor signaling pathway	ATP2B4/ARRDC3
regulation of cytoplasmic translational elongation	CPEB2/CPEB3
negative regulation of bone development	FBXW7/FBN1
positive regulation of cytosolic calcium ion concentration	JPH2/ATP2B4/MYO5A/ITGAV/ITPR1/PRKCE/UBASH3B/PTGER4
cellular response to extracellular stimulus	AXL/DSC2/ITGA6/BMPR2/CLEC16A/SESN3/CPEB4
regulation of protein kinase B signaling	AXL/PLEKHA1/KLF4/MFHAS1/PTPRJ/SESN3/PTEN
ERK1 and ERK2 cascade	NPNT/KLF4/FBXW7/MFHAS1/DUSP10/ITGAV/PTEN/PTGER4
response to acid chemical	IPO5/ATP2B4/SESN3/CPEB4/CPEB3
regulation of heart contraction	DSC2/ATP2B4/ASPH/ATP2A2/ADRB1/ITPR1/KCND2
regulation of release of sequestered calcium ion into cytosol	JPH2/MYO5A/PRKCE/UBASH3B
toll-like receptor 4 signaling pathway	APPL1/MFHAS1/PRKCE
central nervous system neuron development	NFIB/MYCBP2/PTEN/ROBO2
negative regulation of muscle adaptation	ATP2B4/KLF4
locomotion involved in locomotory behavior	ARRDC3/MYO5A
negative regulation of neurotransmitter secretion	PPP1R9A/FMR1
positive regulation of nuclear-transcribed mRNA poly(A) tail shortening	TOB1/CPEB3
negative regulation of sodium ion transmembrane transporter activity	NEDD4L/PRKCE
regulation of osteoclast development	FBXW7/FBN1
camera-type eye development	ATP2B4/KLF4/PDS5B/SLC17A6/BMPR2/FBN1/MAN2A1/GPM6A
dephosphorylation	NPNT/MFHAS1/MTMR9/PTPRJ/SGPP1/DUSP10/PTPRD/UBASH3B/PTEN/PCDH11X
cellular divalent inorganic cation homeostasis	JPH2/ATP2B4/MYO5A/SLC30A7/ATP2A2/ITGAV/ITPR1/PRKCE/UBASH3B/PTGER4
negative regulation of kinase activity	IPO5/PTPRJ/DUSP10/SOCS5/KAT2B/UBASH3B/PTEN
vesicle organization	RAB14/INSIG1/CD2AP/PIKFYVE/WASL/STX17/CUL3/SYT1
post-embryonic animal morphogenesis	BCL2L11/FBN1
cellular anion homeostasis	SLC12A5/SLC12A2
cellular monovalent inorganic anion homeostasis	SLC12A5/SLC12A2
regulation of pinocytosis	AXL/APPL1
regulation of postsynaptic cytosolic calcium ion concentration	MYO5A/ITPR1
negative regulation of sodium ion transmembrane transport	NEDD4L/PRKCE
positive regulation of protein modification by small protein conjugation or removal	ARRDC3/FBXW7/MYCBP2/PTEN/CUL3
positive regulation of dendritic spine development	PPP1R9A/CPEB3/FMR1
regulation of axon guidance	MYCBP2/BMPR2/SEMA6D
regulation of postsynaptic membrane potential	KCNA1/ADRB1/PTEN/PPP1R9A/KCND2
phospholipid dephosphorylation	MTMR9/SGPP1/PTEN
ventricular septum morphogenesis	SOX4/BMPR2/ROBO2
proteasome-mediated ubiquitin-dependent protein catabolic process	CD2AP/FBXW7/HERC2/SOCS5/CLEC16A/EDEM1/NEDD4L/CUL3/WWP2
SREBP signaling pathway	INSIG1/FBXW7
chloride ion homeostasis	SLC12A5/SLC12A2
regulation of nuclear-transcribed mRNA poly(A) tail shortening	TOB1/CPEB3
cellular response to ethanol	PRKCE/PTEN
regulation of adenylate cyclase-activating G protein-coupled receptor signaling pathway	ATP2B4/ARRDC3
regulation of long-term synaptic depression	PPP1R9A/FMR1
dendrite morphogenesis	WASL/NEDD4L/PTPRD/PTEN/PPP1R9A
regulation of amine transport	PER2/SLC6A1/SLC12A2/SYT1
roof of mouth development	PLEKHA1/INSIG1/ASPH/SATB2
cellular response to alcohol	KLF4/PRKCE/PTEN/PTGER4
negative regulation of cation transmembrane transport	NEDD4L/PRKCE/PTEN/FMR1
negative regulation of cell migration	ATP2B4/KLF4/PTPRJ/WASL/DUSP10/PTEN/PTGER4/SEMA6D
cell-cell adhesion via plasma-membrane adhesion molecules	DSC2/CNTN4/KLF4/PALLD/PTPRD/ROBO2/PCDH11X
protein kinase B signaling	AXL/PLEKHA1/KLF4/MFHAS1/PTPRJ/SESN3/PTEN
regulation of endocytosis	AXL/APPL1/CD2AP/WASL/NEDD4L/FMR1

Predicted miR-25 Target Genes and Their Characteristics

Description	geneID
regulation of developmental growth	BCL2L11/DUSP10/ADRB1/BMPR2/NEDD4L/PTEN/SEMA6D/SYT1
negative regulation of protein serine/threonine kinase activity	IPO5/PTPRJ/DUSP10/KAT2B/PTEN
negative chemotaxis	ITGAV/SEMA6D/ROBO2
negative regulation of response to wounding	DUSP10/SLC12A2/UBASH3B/PTEN
protein localization to plasma membrane	FCHO2/ATP2B4/APPL1/MYO5A/DPP10/PRKCE/EXOC5
positive regulation of cellular protein catabolic process	FBXW7/SOCS5/EDEM1/PTEN/FMR1
glycerolipid metabolic process	PLEKHA1/RAB14/INSIG1/FBXW7/MTMR9/PIKFYVE/SOCS6/SOCS5/PTEN
appendage morphogenesis	BCL2L11/ASPH/HOXD10/SOX4/AFF3
limb morphogenesis	BCL2L11/ASPH/HOXD10/SOX4/AFF3
regulation of cytosolic calcium ion concentration	JPH2/ATP2B4/MYO5A/ITGAV/ITPR1/PRKCE/UBASH3B/PTGER4
negative regulation of neurotransmitter transport	PPP1R9A/FMR1
negative regulation of calcineurin-NFAT signaling cascade	ATP2B4/DYRK2
cellular response to sterol depletion	INSIG1/FBXW7
negative regulation of calcineurin-mediated signaling	ATP2B4/DYRK2
synaptic transmission, GABAergic	SLC6A1/PRKCE/PTEN
regulation of wound healing	DUSP10/PRKCE/SLC12A2/UBASH3B/PTEN
regulation of dendrite morphogenesis	NEDD4L/PTPRD/PTEN/PPP1R9A
regulation of phospholipid metabolic process	KLF4/MTMR9/SOCS6/SOCS5
cell-substrate adhesion	AXL/NPNT/BCL2L11/ITGA6/PTPRJ/ITGAV/PRKCE/PTEN
regulation of calcium ion transmembrane transport	JPH2/MYO5A/PRKCE/UBASH3B/FMR1
negative regulation of GTPase activity	IPO5/CPEB2/IQGAP2
G1/S transition of mitotic cell cycle	KLF4/APPL1/SOX4/PTEN/RPS6KB1/CUL3/RBL2
muscle contraction	DSC2/NPNT/ACTC1/ATP2B4/ASPH/KCNA1/ATP2A2/NEDD4L
cellular response to decreased oxygen levels	CPEB2/FMN2/PRKCE/CPEB4/PTEN/KCND2
post-translational protein modification	KBTBD8/TULP4/FBXW7/SOCS6/SOCS5/ASB7/FBN1/CUL3
regulation of phagocytosis	APPL1/PTPRJ/ITGAV/PTEN
neuron recognition	CNTN4/PALLD/ROBO2
brown fat cell differentiation	ITGA6/DUSP10/ADRB1
presynapse assembly	BSN/PTPRD/PTEN
BMP signaling pathway	TRIM33/NEO1/TOB1/BMPR2/FBN1
regulation of leukocyte differentiation	AXL/FBXW7/SOX4/DUSP10/SOCS5/FBN1/UBASH3B
negative regulation of cell motility	ATP2B4/KLF4/PTPRJ/WASL/DUSP10/PTEN/PTGER4/SEMA6D
atrial septum morphogenesis	SOX4/BMPR2
dendrite self-avoidance	CNTN4/PALLD
cellular response to prostaglandin E stimulus	PRKCE/PTGER4
regulation of ribosome biogenesis	KAT2B/PTEN
dicarboxylic acid transport	PER2/SLC17A6/SLC12A2/SYT1
amino acid transport	PER2/SLC17A6/SLC6A1/SLC12A2/SYT1
response to peptide hormone	CPEB2/INSIG1/APPL1/MYO5A/FBN1/KAT2B/SESN3/PTEN/RPS6KB1
amine transport	PER2/SLC6A1/SLC12A2/SYT1
actin filament bundle assembly	MYO1B/FMN2/PTGER4/PPP1R9A/CUL3
negative regulation of myeloid leukocyte differentiation	FBXW7/FBN1/UBASH3B
apoptotic cell clearance	AXL/RAB14/ITGAV
regulation of pri-miRNA transcription by RNA polymerase II	TEAD1/KLF4/NFIB
heart valve morphogenesis	SOX4/BMPR2/ROBO2
negative regulation of BMP signaling pathway	TRIM33/TOB1/FBN1
regulation of transcription regulatory region DNA binding	KLF4/FBXW7/PER2
regulation of protein stability	ASPH/FBXW7/MTMR9/SOX4/NEDD4L/PTEN/CUL3
negative regulation of transferase activity	IPO5/PTPRJ/DUSP10/SOCS5/KAT2B/UBASH3B/PTEN
regulation of proteolysis involved in cellular protein catabolic process	FBXW7/SOCS5/CLEC16A/EDEM1/PTEN/FMR1
regulation of calcium ion transport into cytosol	JPH2/MYO5A/PRKCE/UBASH3B
N-terminal protein amino acid acetylation	SOX4/KAT2B
response to sterol depletion	INSIG1/FBXW7

Predicted miR-25 Target Genes and Their Characteristics

Description	geneID
negative regulation of phosphatidylinositol 3-kinase signaling	KLF4/PTEN
heat generation	ARRDC3/ADRB1
negative regulation of chemokine production	KLF4/SOCS5
maintenance of synapse structure	ERC2/BSN
Golgi vesicle transport	GOLGA3/RAB14/INSIG1/MYO1B/MYO5A/STX17/EXOC5/CUL3
tissue migration	ACTC1/ATP2B4/KLF4/FBXW7/DUSP10/BMPR2/PRKCE/PTEN
regulation of protein localization to plasma membrane	ATP2B4/APPL1/DPP10/PRKCE
actin filament bundle organization	MYO1B/FMN2/PTGER4/PPP1R9A/CUL3
phospholipid metabolic process	PLEKHA1/RAB14/KLF4/MTMR9/PIKFYVE/SGPP1/SOCS6/SOCS5/PTEN
pri-miRNA transcription by RNA polymerase II	TEAD1/KLF4/NFIB
presynapse organization	BSN/PTPRD/PTEN
cellular calcium ion homeostasis	JPH2/ATP2B4/MYO5A/ATP2A2/ITGAV/ITPR1/PRKCE/UBASH3B/PTGER4
cytoplasmic translation	CPEB2/CPEB4/CPEB3/FMR1
negative regulation of ion transmembrane transport	NEDD4L/PRKCE/PTEN/FMR1
regulation of epithelial cell migration	ATP2B4/KLF4/FBXW7/DUSP10/BMPR2/PRKCE/PTEN
regulation of synapse organization	PTPRD/PTEN/GPM6A/PPP1R9A/ROBO2/CTTNBP2
cellular response to starvation	DSC2/BMPR2/CLEC16A/SESN3/CPEB4
regulation of ubiquitin-protein transferase activity	ARRDC3/FBXW7/PTEN
cell-matrix adhesion	NPNT/BCL2L11/ITGA6/PTPRJ/ITGAV/PTEN
extrinsic apoptotic signaling pathway	BCL2L11/ITGA6/APPL1/SGPP1/ITGAV/PTEN
post-Golgi vesicle-mediated transport	RAB14/MYO1B/MYO5A/EXOC5
cellular sodium ion homeostasis	NEDD4L/SLC12A2
negative regulation of sodium ion transport	NEDD4L/PRKCE
wound healing, spreading of epidermal cells	PTEN/COL5A1
osteoclast development	FBXW7/FBN1
postsynaptic density assembly	PTPRD/PTEN
positive regulation of nuclear-transcribed mRNA catabolic process, deadenylation-dependent decay	TOB1/CPEB3
osteoblast differentiation	NPNT/MEF2D/IBSP/TOB1/SATB2/BMPR2
positive regulation of lymphocyte differentiation	AXL/SOX4/DUSP10/SOCS5
negative regulation of leukocyte differentiation	FBXW7/SOCS5/FBN1/UBASH3B
endochondral bone morphogenesis	MEF2D/BMPR2/MMP16
phagocytosis	AXL/RAB14/APPL1/PTPRJ/WASL/ITGAV/PRKCE/PTEN
regulation of neurotransmitter secretion	ATP2A2/PPP1R9A/SYT1/FMR1
fat cell differentiation	INSIG1/ITGA6/KLF4/PER2/DUSP10/ADRB1
cellular response to oxygen levels	CPEB2/FMN2/PRKCE/CPEB4/PTEN/KCND2
homophilic cell adhesion via plasma membrane adhesion molecules	DSC2/CNTN4/PALLD/ROBO2/PCDH11X
response to BMP	TRIM33/NEO1/TOB1/BMPR2/FBN1
cellular response to BMP stimulus	TRIM33/NEO1/TOB1/BMPR2/FBN1
calcium ion homeostasis	JPH2/ATP2B4/MYO5A/ATP2A2/ITGAV/ITPR1/PRKCE/UBASH3B/PTGER4
chondrocyte proliferation	BMPR2/MMP16
regulation of Notch signaling pathway	FBXW7/KAT2B/ROBO2/WWP2
regulation of cellular response to growth factor stimulus	NPNT/TRIM33/ATP2B4/NEO1/TOB1/BMPR2/FBN1
protein localization to extracellular region	CD2AP/MYO5A/PER2/SOX4/FBN1/ITPR1/PRKCE/SLC12A2/PTGER4
phosphatidylinositol phosphorylation	PIKFYVE/SOCS6/SOCS5
inositol phosphate-mediated signaling	ATP2B4/ITPR1/DYRK2
regulation of action potential	DSC2/ATP2A2/FMR1
striated muscle tissue development	JPH2/ACTC1/MAP2K4/MEF2D/FOXN2/HOXD10/COL19A1/PTEN
regulation of phosphatidylinositol 3-kinase activity	KLF4/SOCS6/SOCS5
positive regulation of proteasomal protein catabolic process	FBXW7/SOCS5/EDEM1/FMR1
positive regulation of synaptic transmission	PRKCE/PTEN/PPP1R9A/SYT1/FMR1
negative regulation of systemic arterial blood pressure	ADRB1/BMPR2
regulation of nuclear-transcribed mRNA catabolic process, deadenylation-dependent decay	TOB1/CPEB3
positive regulation of neuron apoptotic process	BCL2L11/MAP2K4/FBXW7

Predicted miR-25 Target Genes and Their Characteristics

Description	geneID
platelet-derived growth factor receptor signaling pathway	PLEKHA1/PTPRJ/PTEN
vacuole organization	RAB14/RAB23/PIKFYVE/STX17/MAN2A1
neurotransmitter secretion	ERC2/ATP2A2/PPP1R9A/SYT1/FMR1
signal release from synapse	ERC2/ATP2A2/PPP1R9A/SYT1/FMR1
ameboidal-type cell migration	ATP2B4/KLF4/APPL1/FBXW7/DUSP10/BMPR2/PRKCE/PTEN/SEMA6D
plasma membrane organization	WASL/ATP2A2/PTEN/COL5A1
axonogenesis	CNTN4/PALLD/NEO1/NFIB/MYCBP2/BMPR2/PTEN/SEMA6D/ROBO2
negative regulation of cellular component movement	ATP2B4/KLF4/PTPRJ/WASL/DUSP10/PTEN/PTGER4/SEMA6D
response to amino acid	IPO5/SESN3/CPEB4/CPEB3
pulmonary valve development	BMPR2/ROBO2
regulation of translational elongation	CPEB2/CPEB3
positive regulation of amino acid transport	SLC6A1/SLC12A2
cellular response to fluid shear stress	KLF4/SOCS5
postsynaptic specialization assembly	PTPRD/PTEN
negative regulation of transcription regulatory region DNA binding	FBXW7/PER2
heart valve development	SOX4/BMPR2/ROBO2
positive regulation of protein localization to plasma membrane	ATP2B4/DPP10/PRKCE
negative regulation of catabolic process	ATP2B4/MTMR9/TOB1/MYCBP2/CLEC16A/FMN2/FMR1
regulation of cell cycle phase transition	ATP2B4/FOXN3/KLF4/APPL1/FBXW7/SOX4/PTEN/CUL3/RBL2
negative regulation of developmental growth	DUSP10/ADRB1/PTEN/SEMA6D
positive regulation of cellular carbohydrate metabolic process	KAT2B/PRKCE/DYRK2
heterotypic cell-cell adhesion	DSC2/KLF4/ITGAV
dendritic spine morphogenesis	WASL/PTEN/PPP1R9A
negative regulation of cellular protein localization	INSIG1/RAB23/MFHAS1/NEDD4L
regulation of actin filament-based process	DSC2/CD2AP/WASL/ATP2A2/PRKCE/PTGER4/PPP1R9A/IQGAP2
muscle organ development	JPH2/ACTC1/MEF2D/FOXN2/HOXD10/ZFHX3/COL19A1/PTEN
melanin biosynthetic process	APPL1/MYO5A
negative regulation of phagocytosis	APPL1/PTEN
cellular response to prostaglandin stimulus	PRKCE/PTGER4
liver morphogenesis	FBXW7/CUL3
regulation of cell size	SLC12A5/BMPR2/SLC12A2/PTEN/SEMA6D
muscle tissue development	JPH2/ACTC1/MAP2K4/MEF2D/FOXN2/HOXD10/COL19A1/PTEN
negative regulation of growth	PTPRJ/DUSP10/ADRB1/BMPR2/PTEN/SEMA6D
regulation of transmembrane receptor protein serine/threonine kinase signaling pathway	NPNT/TRIM33/NEO1/TOB1/BMPR2/FBN1
regulation of plasma membrane bounded cell projection assembly	WASL/ATP8B1/GPM6A/PPP1R9A/FMR1
ossification	NPNT/MEF2D/IBSP/TOB1/SATB2/BMPR2/PTGER4/MMP16
regulation of muscle system process	DSC2/NPNT/ATP2B4/KLF4/KCNA1/ATP2A2
atrial septum development	SOX4/BMPR2
melanin metabolic process	APPL1/MYO5A
pinocytosis	AXL/APPL1
L-glutamate transmembrane transport	PER2/SLC17A6
calcium ion-regulated exocytosis of neurotransmitter	ATP2A2/SYT1
positive regulation of dendrite extension	NEDD4L/SYT1
regulation of cell projection assembly	WASL/ATP8B1/GPM6A/PPP1R9A/FMR1
regulation of G1/S transition of mitotic cell cycle	KLF4/APPL1/SOX4/PTEN/RBL2
regulation of sodium ion transmembrane transport	ATP2B4/NEDD4L/PRKCE
regulation of cell morphogenesis	MYCBP2/BMPR2/NEDD4L/PTPRD/PTEN/SEMA6D/PPP1R9A/ROBO2/SYT1
memory	SLC6A1/PTEN/ATXN1/CPEB3
synaptic vesicle exocytosis	ERC2/ATP2A2/SYT1/FMR1
regulation of axonogenesis	MYCBP2/BMPR2/PTEN/SEMA6D/ROBO2
circadian regulation of gene expression	PER2/MYCBP2/ZFHX3
positive regulation of cell-substrate adhesion	NPNT/ITGA6/PTPRJ/PRKCE
regulation of circadian rhythm	FBXW7/PER2/ADRB1/ZFHX3

Predicted miR-25 Target Genes and Their Characteristics

Description	geneID
regulation of cellular protein catabolic process	FBXW7/SOCS5/CLEC16A/EDEM1/PTEN/FMR1
positive regulation of cellular protein localization	IPO5/ATP2B4/CD2AP/FBXW7/DPP10/EDEM1/PRKCE
actin-mediated cell contraction	DSC2/ACTC1/ATP2A2/NEDD4L
regulation of protein localization to cell periphery	ATP2B4/APPL1/DPP10/PRKCE
atrioventricular valve morphogenesis	SOX4/BMPR2
endoplasmic reticulum calcium ion homeostasis	ATP2A2/ITPR1
regulation of toll-like receptor 4 signaling pathway	APPL1/MFHAS1
response to prostaglandin E	PRKCE/PTGER4
fibroblast apoptotic process	BCL2L11/CUL3
secondary metabolite biosynthetic process	APPL1/MYO5A
regulation of organic acid transport	PER2/SLC6A1/SLC12A2
regulation of osteoclast differentiation	FBXW7/FBN1/UBASH3B
gastrulation	KLF4/ITGAV/BMPR2/COL5A1/CUL3
connective tissue development	MEF2D/ARRDC3/NFIB/SATB2/BMPR2/COL5A1
negative regulation of epithelial cell migration	ATP2B4/KLF4/DUSP10/PTEN
positive regulation of secretion	CD2AP/SLC6A1/SOX4/PRKCE/SLC12A2/PTGER4/SYT1
protein localization to cell periphery	FCHO2/ATP2B4/APPL1/MYO5A/DPP10/PRKCE/EXOC5
positive regulation of protein localization to cell periphery	ATP2B4/DPP10/PRKCE
regulation of synaptic plasticity	CNTN4/PTEN/PPP1R9A/CPEB3/FMR1
glycerophospholipid metabolic process	PLEKHA1/RAB14/MTMR9/PIKFYVE/SOCS6/SOCS5/PTEN
negative regulation of G1/S transition of mitotic cell cycle	KLF4/SOX4/PTEN/RBL2
regulation of dendrite extension	NEDD4L/SYT1
negative regulation of synapse organization	PTEN/ROBO2
ovulation cycle	AXL/PLEKHA1/ROBO2
regulation of proteasomal protein catabolic process	FBXW7/SOCS5/CLEC16A/EDEM1/FMR1
formation of primary germ layer	KLF4/ITGAV/BMPR2/COL5A1
regulation of protein localization to nucleus	IPO5/RAB23/CD2AP/MFHAS1
regulation of behavior	ARRDC3/ADRB1/ZFHX3
regulation of autophagy	BCL2L11/FBXW7/MTMR9/PIKFYVE/CLEC16A/ITPR1/SESN3
negative regulation of transmembrane transport	NEDD4L/PRKCE/PTEN/FMR1
regulation of lipid metabolic process	INSIG1/KLF4/FBXW7/MTMR9/SOCS6/SOCS5/PRKCE/RBL2
negative regulation of inflammatory response	KLF4/MFHAS1/DUSP10/SOCS5/PTGER4
glycerolipid biosynthetic process	PLEKHA1/RAB14/FBXW7/MTMR9/PIKFYVE/PTEN
B cell apoptotic process	BCL2L11/PTEN
atrioventricular valve development	SOX4/BMPR2
central nervous system projection neuron axonogenesis	NFIB/MYCBP2
negative regulation of cAMP-mediated signaling	ATP2B4/ARRDC3
monovalent inorganic anion homeostasis	SLC12A5/SLC12A2
transepithelial transport	ITPR1/SLC12A2
regulation of bone development	FBXW7/FBN1
excitatory synapse assembly	PTPRD/PTEN
cell adhesion mediated by integrin	NPNT/ITGAV/FBN1
cellular polysaccharide biosynthetic process	B3GALT2/PER2/DYRK2
carboxylic acid transport	PER2/SLC17A6/SLC6A1/ATP8B1/SLC12A2/RPS6KB1/SYT1
lipid modification	MTMR9/PIKFYVE/SGPP1/SOCS6/SOCS5/PTEN
cell junction assembly	ITGA6/PTPRJ/BSN/PTPRD/PTEN/GPM6A/PPP1R9A/ROBO2
regulation of protein secretion	CD2AP/PER2/SOX4/ITPR1/PRKCE/SLC12A2/PTGER4
transmission of nerve impulse	KCNA1/KCND2/FMR1
lipid phosphorylation	PIKFYVE/SOCS6/SOCS5
organic acid transport	PER2/SLC17A6/SLC6A1/ATP8B1/SLC12A2/RPS6KB1/SYT1
positive regulation of protein transport	IPO5/CD2AP/FBXW7/SOX4/EDEM1/PRKCE/PTGER4
outflow tract septum morphogenesis	BMPR2/ROBO2
actomyosin structure organization	ACTC1/CDC42BPA/PTGER4/PPP1R9A/CUL3

Predicted miR-25 Target Genes and Their Characteristics

Description	geneID
regulation of cell junction assembly	PTPRJ/PTPRD/PTEN/PPP1R9A/ROBO2
negative regulation of cell cycle G1/S phase transition	KLF4/SOX4/PTEN/RBL2
protein localization to nucleus	IPO5/RAB23/APPL1/CD2AP/MFHAS1/PIKFYVE
cardiac muscle cell contraction	DSC2/ATP2A2/NEDD4L
viral entry into host cell	AXL/PIKFYVE/ITGAV/WWP2
transmembrane receptor protein serine/threonine kinase signaling pathway	NPNT/TRIM33/APPL1/NEO1/TOB1/BMPR2/FBN1
positive regulation of heart rate	ATP2A2/ADRB1
negative regulation of protein phosphorylation	IPO5/KLF4/PTPRJ/DUSP10/SOCS5/KAT2B/UBASH3B/PTEN
signal transduction in absence of ligand	BCL2L11/APPL1/ITGAV
cardiac muscle cell action potential	DSC2/ATP2A2/NEDD4L
extrinsic apoptotic signaling pathway in absence of ligand	BCL2L11/APPL1/ITGAV
vesicle cytoskeletal trafficking	MYO1B/MYO5A/WASL
positive regulation of translation	SOX4/RPS6KB1/CPEB3/FMR1
regulation of alternative mRNA splicing, via spliceosome	NOVA1/QKI/FMR1
ventricular septum development	SOX4/BMPR2/ROBO2
response to starvation	DSC2/BMPR2/CLEC16A/SESN3/CPEB4
myelination	MYO5A/PIKFYVE/QKI/PTEN
endochondral ossification	MEF2D/MMP16
protein deglycosylation	EDEM1/MAN2A1
N-terminal protein amino acid modification	SOX4/KAT2B
replacement ossification	MEF2D/MMP16
regulation of axon regeneration	KLF4/PTEN
potassium ion homeostasis	SLC12A5/SLC12A2
regulation of protein exit from endoplasmic reticulum	INSIG1/EDEM1
negative regulation of potassium ion transmembrane transporter activity	NEDD4L/PTEN
epithelial cell migration	ATP2B4/KLF4/FBXW7/DUSP10/BMPR2/PRKCE/PTEN
cardiac septum morphogenesis	SOX4/BMPR2/ROBO2
cellular response to hypoxia	CPEB2/FMN2/PRKCE/PTEN/KCND2
immune response-regulating cell surface receptor signaling pathway involved in phagocytosis	APPL1/PTPRJ/WASL/PRKCE
ensheathment of neurons	MYO5A/PIKFYVE/QKI/PTEN
axon ensheathment	MYO5A/PIKFYVE/QKI/PTEN
Fc-gamma receptor signaling pathway involved in phagocytosis	APPL1/PTPRJ/WASL/PRKCE
polysaccharide biosynthetic process	B3GALT2/PER2/DYRK2
lymphocyte differentiation	AXL/PTPRJ/SOX4/DUSP10/SOCS5/PTGER4/KLF6
epithelium migration	ATP2B4/KLF4/FBXW7/DUSP10/BMPR2/PRKCE/PTEN
cardiac muscle contraction	DSC2/ACTC1/ATP2A2/NEDD4L
cardiac atrium morphogenesis	SOX4/BMPR2
cell volume homeostasis	SLC12A5/SLC12A2
regulation of epidermal growth factor-activated receptor activity	FBXW7/SOCS5
positive regulation of proteolysis	BCL2L11/ASPH/FBXW7/SOCS5/EDEM1/PTEN/FMR1
positive regulation of establishment of protein localization	IPO5/CD2AP/FBXW7/SOX4/EDEM1/PRKCE/PTGER4
negative regulation of ERK1 and ERK2 cascade	KLF4/DUSP10/PTEN
response to estradiol	STRN3/BCL2L11/SLC6A1/PTEN
response to decreased oxygen levels	CPEB2/FMN2/ITPR1/PRKCE/CPEB4/PTEN/KCND2
interaction with host	AXL/BCL2L11/PIKFYVE/ITGAV/WWP2
mesenchyme development	KBTBD8/ACTC1/BMPR2/PTEN/SEMA6D/ROBO2
intrinsic apoptotic signaling pathway	BCL2L11/FBXW7/SGPP1/ITPR1/CUL3/DYRK2
Fc-gamma receptor signaling pathway	APPL1/PTPRJ/WASL/PRKCE
insulin secretion	MYO5A/PER2/SOX4/ITPR1/PRKCE
negative regulation of protein ubiquitination	PER2/SOX4/PRKCE
regulation of synaptic vesicle exocytosis	ATP2A2/SYT1/FMR1
muscle hypertrophy in response to stress	ATP2B4/ATP2A2
cardiac muscle adaptation	ATP2B4/ATP2A2

Predicted miR-25 Target Genes and Their Characteristics

Description	geneID
cardiac muscle hypertrophy in response to stress	ATP2B4/ATP2A2
phosphatidylinositol dephosphorylation	MTMR9/PTEN
face morphogenesis	PLEKHA1/ASPH
postsynapse assembly	PTPRD/PTEN
negative regulation of protein localization to nucleus	RAB23/MFHAS1
regulation of neuron apoptotic process	AXL/BCL2L11/MAP2K4/FBXW7/CPEB4
peptidyl-tyrosine phosphorylation	AXL/MAP2K4/FBXW7/PTPRJ/SOCS5/PRKCE/DYRK2
hippocampus development	ATP2B4/KCNA1/PTEN
protein secretion	CD2AP/MYO5A/PER2/SOX4/ITPR1/PRKCE/SLC12A2/PTGER4
cold-induced thermogenesis	NOVA1/ARRDC3/PER2/ADRB1
regulation of cold-induced thermogenesis	NOVA1/ARRDC3/PER2/ADRB1
establishment of protein localization to extracellular region	CD2AP/MYO5A/PER2/SOX4/ITPR1/PRKCE/SLC12A2/PTGER4
cellular carbohydrate metabolic process	B3GALT2/PER2/KAT2B/PRKCE/PTEN/DYRK2
peptidyl-tyrosine modification	AXL/MAP2K4/FBXW7/PTPRJ/SOCS5/PRKCE/DYRK2
Fc receptor mediated stimulatory signaling pathway	APPL1/PTPRJ/WASL/PRKCE
regulation of cell-substrate adhesion	NPNT/ITGA6/PTPRJ/PRKCE/PTEN
aortic valve development	BMPR2/ROBO2
response to prostaglandin	PRKCE/PTGER4
regulation of axon extension involved in axon guidance	BMPR2/SEMA6D
regulation of neuron projection regeneration	KLF4/PTEN
postsynaptic density organization	PTPRD/PTEN
neurotransmitter reuptake	PER2/SLC6A1
negative regulation of cell junction assembly	PTEN/ROBO2
vasculogenesis	FBXW7/ITGAV/QKI
regulation of peptide secretion	CD2AP/PER2/SOX4/ITPR1/PRKCE/SLC12A2/PTGER4
adenylate cyclase-activating G protein-coupled receptor signaling pathway	ATP2B4/ARRDC3/ADRB1/PTGER4
entry into host	AXL/PIKFYVE/ITGAV/WWP2
negative regulation of translation	CPEB2/TOB1/CPEB4/CPEB3/FMR1
positive regulation of carbohydrate metabolic process	KAT2B/PRKCE/DYRK2
dendritic spine organization	WASL/PTEN/PPP1R9A
cell junction maintenance	ERC2/BSN
wound healing, spreading of cells	PTEN/COL5A1
epiboly involved in wound healing	PTEN/COL5A1
negative regulation of potassium ion transmembrane transport	NEDD4L/PTEN
cellular response to nutrient levels	DSC2/BMPR2/CLEC16A/SESN3/CPEB4
response to nutrient levels	DSC2/ADRB1/BMPR2/CLEC16A/SESN3/CPEB4/PTEN/RPS6KB1
retina development in camera-type eye	ATP2B4/BMPR2/MAN2A1/GPM6A
regulation of fibroblast migration	APPL1/PRKCE
positive regulation of phosphatase activity	NPNT/MTMR9
long-chain fatty acid biosynthetic process	MYO5A/QKI
response to arsenic-containing substance	CPEB2/PTEN
epiboly	PTEN/COL5A1
regulation of cardiac muscle cell action potential	DSC2/ATP2A2
postsynaptic specialization organization	PTPRD/PTEN
cellular carbohydrate biosynthetic process	B3GALT2/PER2/DYRK2
negative regulation of protein transport	INSIG1/RAB23/PTGER4/WWP2
regulation of mRNA processing	NOVA1/QKI/CPEB3/FMR1
alternative mRNA splicing, via spliceosome	NOVA1/QKI/FMR1
regulation of ERK1 and ERK2 cascade	NPNT/KLF4/FBXW7/MFHAS1/DUSP10/PTEN
forebrain development	AXL/ATP2B4/KCNA1/NFIB/SATB2/PTEN/ROBO2
spinal cord motor neuron differentiation	HOXD10/SOX4
positive regulation of BMP signaling pathway	NEO1/BMPR2
regulation of calcineurin-NFAT signaling cascade	ATP2B4/DYRK2

Predicted miR-25 Target Genes and Their Characteristics

Description	geneID
bone cell development	FBXW7/FBN1
regulation of GTPase activity	DOCK9/IPO5/CPEB2/ITGA6/GIT2/ADRB1/EVI5/IQGAP2
regulation of sodium ion transport	ATP2B4/NEDD4L/PRKCE
vesicle localization	MYO1B/MYO5A/WASL/PTEN/CUL3
negative regulation of establishment of protein localization	INSIG1/RAB23/PTGER4/WWP2
adaptive thermogenesis	NOVA1/ARRDC3/PER2/ADRB1
regulation of calcium ion transmembrane transporter activity	JPH2/MYO5A/FMR1
negative regulation of phosphorylation	IPO5/KLF4/PTPRJ/DUSP10/SOCS5/KAT2B/UBASH3B/PTEN
peptidyl-serine phosphorylation	ATP2B4/MARK1/PRKCE/PTEN/RPS6KB1/DYRK2
cellular response to oxidative stress	AXL/PLEKHA1/CPEB2/KLF4/FBXW7/ATP2A2
glycerophospholipid biosynthetic process	PLEKHA1/RAB14/MTMR9/PIKFYVE/PTEN
response to oxygen levels	CPEB2/FMN2/ITPR1/PRKCE/CPEB4/PTEN/KCND2
nuclear-transcribed mRNA poly(A) tail shortening	TOB1/CPEB3
cardiac atrium development	SOX4/BMPR2
response to fluid shear stress	KLF4/SOCS5
head morphogenesis	PLEKHA1/ASPH
regulation of transforming growth factor beta production	CD2AP/ITGAV
dendrite extension	NEDD4L/SYT1
regulation of calcineurin-mediated signaling	ATP2B4/DYRK2
regulation of epidermal growth factor receptor signaling pathway	FBXW7/PTPRJ/SOCS5
negative regulation of ion transport	NEDD4L/PRKCE/PTEN/FMR1
positive regulation of secretion by cell	CD2AP/SOX4/PRKCE/SLC12A2/PTGER4/SYT1
response to carbohydrate	MAP2K4/SLC6A1/SOX4/PRKCE/PTEN
skeletal muscle tissue development	MEF2D/FOXN2/HOXD10/COL19A1
platelet activation	AXL/ITPR1/PRKCE/UBASH3B
negative regulation of hemopoiesis	FBXW7/SOCS5/FBN1/UBASH3B
hindlimb morphogenesis	HOXD10/AFF3
axon extension involved in axon guidance	BMPR2/SEMA6D
neuron projection extension involved in neuron projection guidance	BMPR2/SEMA6D
semi-lunar valve development	BMPR2/ROBO2
anion transmembrane transport	SLC9A2/PER2/SLC12A5/SLC17A6/SLC6A1/SLC12A2
response to alcohol	ACTC1/KLF4/PRKCE/PTEN/PTGER4
monovalent inorganic cation homeostasis	SLC9A2/SLC12A5/NEDD4L/SLC12A2
positive regulation of leukocyte differentiation	AXL/SOX4/DUSP10/SOCS5
regulation of ubiquitin-dependent protein catabolic process	FBXW7/SOCS5/CLEC16A/PTEN
positive regulation of T cell differentiation	SOX4/DUSP10/SOCS5
neuron projection organization	WASL/PTEN/PPP1R9A
negative regulation of protein modification by small protein conjugation or removal	PER2/SOX4/PRKCE
positive regulation of muscle cell apoptotic process	MAP2K4/PTEN
transforming growth factor beta production	CD2AP/ITGAV
ventricular cardiac muscle cell action potential	DSC2/NEDD4L
regulation of TORC1 signaling	CLEC16A/SESN3
negative regulation of blood circulation	ATP2A2/BMPR2
positive regulation of response to endoplasmic reticulum stress	BCL2L11/EDEM1
developmental cell growth	MAP2K4/BMPR2/NEDD4L/SEMA6D/SYT1
negative regulation of cellular amide metabolic process	CPEB2/TOB1/CPEB4/CPEB3/FMR1
regulation of hemopoiesis	AXL/FBXW7/SOX4/DUSP10/SOCS5/FBN1/KAT2B/UBASH3B
membrane assembly	PTPRD/PTEN
regulation of actin cytoskeleton reorganization	CD2AP/IQGAP2
negative regulation of myeloid cell differentiation	FBXW7/FBN1/UBASH3B
protein dephosphorylation	MFHAS1/PTPRJ/DUSP10/PTPRD/UBASH3B/PTEN
neuron migration	AXL/MARK1/SATB2/GPM6A
Fc receptor signaling pathway	MAP2K4/APPL1/PTPRJ/WASL/PRKCE

Predicted miR-25 Target Genes and Their Characteristics

Description	geneID
autophagosome assembly	RAB23/PIKFYVE/STX17
positive regulation of cytokinesis	PRKCE/CUL3
positive regulation of organic acid transport	SLC6A1/SLC12A2
Arp2/3 complex-mediated actin nucleation	WASL/IQGAP2
negative regulation of Notch signaling pathway	FBXW7/WWP2
autophagosome maturation	STX17/CLEC16A
apoptotic process involved in development	BCL2L11/ROBO2
positive regulation of pri-miRNA transcription by RNA polymerase II	TEAD1/KLF4
cellular response to topologically incorrect protein	BCL2L11/EDEM1/HERPUD2/CUL3
embryonic skeletal system morphogenesis	HOXD10/SATB2/MMP16
regulation of ERBB signaling pathway	FBXW7/PTPRJ/SOCS5
positive regulation of neuron death	BCL2L11/MAP2K4/FBXW7
cellular response to ketone	KLF4/PRKCE/PTGER4
chloride transmembrane transport	SLC12A5/SLC6A1/SLC12A2
positive regulation of cellular amide metabolic process	SOX4/RPS6KB1/CPEB3/FMR1
neuron apoptotic process	AXL/BCL2L11/MAP2K4/FBXW7/CPEB4
osteoclast differentiation	FBXW7/FBN1/UBASH3B
skeletal muscle organ development	MEF2D/FOXN2/HOXD10/COL19A1
negative regulation of potassium ion transport	NEDD4L/PTEN
lung alveolus development	BMPR2/MAN2A1
regulation of heart morphogenesis	BMPR2/ROBO2
urogenital system development	NPNT/BCL2L11/SOX4/FBN1/PTEN/ROBO2
cell-substrate junction assembly	ITGA6/PTPRJ/PTEN
pigmentation	BCL2L11/MYO5A/PIKFYVE
autophagosome organization	RAB23/PIKFYVE/STX17
movement in host environment	AXL/PIKFYVE/ITGAV/WWP2
cellular response to hydrogen peroxide	AXL/PLEKHA1/KLF4
peptidyl-serine modification	ATP2B4/MARK1/PRKCE/PTEN/RPS6KB1/DYRK2
neurotransmitter uptake	PER2/SLC6A1
positive regulation of glucose metabolic process	KAT2B/DYRK2
positive regulation of actin filament polymerization	WASL/PRKCE/IQGAP2
cellular polysaccharide metabolic process	B3GALT2/PER2/DYRK2
mitotic DNA damage checkpoint	FOXN3/SOX4/RBL2
positive regulation of protein secretion	CD2AP/SOX4/PRKCE/PTGER4
neuron projection extension	BMPR2/NEDD4L/SEMA6D/SYT1
negative regulation of mitotic cell cycle phase transition	FOXN3/KLF4/SOX4/PTEN/RBL2
skin development	DSC2/ITGA6/PALLD/ARRDC3/MYO5A/SGPP1/COL5A1
regulation of muscle contraction	DSC2/NPNT/KCNA1/ATP2A2
postsynapse organization	WASL/PTPRD/PTEN/PPP1R9A
regulation of gastrulation	KLF4/COL5A1
regulation of calcium ion-dependent exocytosis	ATP2A2/SYT1
positive regulation of heart contraction	ATP2A2/ADRB1
regulation of cardiac muscle cell contraction	DSC2/ATP2A2
negative regulation of intracellular protein transport	INSIG1/RAB23
regulation of autophagy of mitochondrion	FBXW7/CLEC16A
regulation of cyclin-dependent protein serine/threonine kinase activity	IPO5/KAT2B/PTEN
regulation of muscle adaptation	ATP2B4/KLF4/ATP2A2
artery development	SOX4/BMPR2/ROBO2
cell-substrate junction organization	ITGA6/PTPRJ/PTEN
positive regulation of ubiquitin-dependent protein catabolic process	FBXW7/SOCS5/PTEN
regulation of calcium ion transport	JPH2/MYO5A/PRKCE/UBASH3B/FMR1
temperature homeostasis	NOVA1/ARRDC3/PER2/ADRB1
regulation of actin filament polymerization	WASL/PRKCE/PPP1R9A/IQGAP2

Predicted miR-25 Target Genes and Their Characteristics

Description	geneID
negative regulation of epithelial cell proliferation	FBXW7/NFIB/DUSP10/PTEN
calcineurin-NFAT signaling cascade	ATP2B4/DYRK2
membrane biogenesis	PTPRD/PTEN
regulation of heart rate	DSC2/ATP2A2/ADRB1
cardiac muscle hypertrophy	ATP2B4/MAP2K4/ATP2A2
positive regulation of plasma membrane bounded cell projection assembly	WASL/GPM6A/FMR1
viral life cycle	AXL/PIKFYVE/ITGAV/NEDD4L/WWP2/FMR1
lung development	FBXW7/NFIB/BMPR2/MAN2A1
peptide hormone secretion	MYO5A/PER2/SOX4/ITPR1/PRKCE
actin cytoskeleton reorganization	CD2AP/CDC42BPA/IQGAP2
regulation of anion transport	PER2/SLC6A1/SLC12A2
regulation of receptor-mediated endocytosis	CD2AP/WASL/FMR1
negative regulation of cell development	CNTN4/FBXW7/DUSP10/FBN1/PTEN/SEMA6D
blood vessel remodeling	AXL/BMPR2
endocardial cushion development	BMPR2/ROBO2
amino acid import	PER2/SLC6A1
phenol-containing compound biosynthetic process	APPL1/MYO5A
clathrin-dependent endocytosis	FCHO2/WASL
heart morphogenesis	ACTC1/SOX4/BMPR2/ROBO2/COL5A1
response to transforming growth factor beta	NPNT/TRIM33/APPL1/FBN1/ZFHX3
striated muscle contraction	DSC2/ACTC1/ATP2A2/NEDD4L
contractile actin filament bundle assembly	PTGER4/PPP1R9A/CUL3
stress fiber assembly	PTGER4/PPP1R9A/CUL3
regulation of cyclin-dependent protein kinase activity	IPO5/KAT2B/PTEN
telencephalon development	ATP2B4/KCNA1/NFIB/PTEN/ROBO2
learning or memory	SLC12A5/SLC6A1/PTEN/ATXN1/CPEB3
striated muscle hypertrophy	ATP2B4/MAP2K4/ATP2A2
regulation of synapse assembly	PTPRD/PPP1R9A/ROBO2
regulation of postsynapse organization	PTPRD/PTEN/PPP1R9A
glycogen biosynthetic process	PER2/DYRK2
glucan biosynthetic process	PER2/DYRK2
fibroblast migration	APPL1/PRKCE
endodermal cell differentiation	ITGAV/COL5A1
TORC1 signaling	CLEC16A/SESN3
developmental pigmentation	BCL2L11/MYO5A
regulation of p38MAPK cascade	MFHAS1/DUSP10
regulation of cell-cell adhesion	ITGA6/KLF4/SOX4/DUSP10/SOCS6/SOCS5/UBASH3B
respiratory tube development	FBXW7/NFIB/BMPR2/MAN2A1
regulation of lymphocyte differentiation	AXL/SOX4/DUSP10/SOCS5
regulation of insulin secretion	PER2/SOX4/ITPR1/PRKCE
chondrocyte differentiation	MEF2D/NFIB/BMPR2
integrin-mediated signaling pathway	ITGA6/ITGAV/CUL3
mitotic DNA integrity checkpoint	FOXN3/SOX4/RBL2
leukocyte apoptotic process	AXL/BCL2L11/PTEN
negative regulation of cellular response to growth factor stimulus	TRIM33/ATP2B4/TOB1/FBN1
ovulation cycle process	PLEKHA1/ROBO2
collagen catabolic process	COL19A1/MMP16
negative regulation of blood pressure	ADRB1/BMPR2
negative regulation of cold-induced thermogenesis	NOVA1/ARRDC3
regulation of actin filament-based movement	DSC2/ATP2A2
regulation of intracellular protein transport	IPO5/INSIG1/RAB23/FBXW7/EDEM1
muscle hypertrophy	ATP2B4/MAP2K4/ATP2A2
spinal cord development	HOXD10/SOX4/ROBO2

Predicted miR-25 Target Genes and Their Characteristics

Description	geneID
limbic system development	ATP2B4/KCNA1/PTEN
negative regulation of cellular catabolic process	ATP2B4/MTMR9/TOB1/CLEC16A/FMR1
vesicle budding from membrane	INSIG1/WASL/CUL3
positive regulation of transmembrane receptor protein serine/threonine kinase signaling pathway	NPNT/NEO1/BMPR2

Supplementary Table S2. Antibodies used for western blot.

Antigen	Species	Manufacturer	Catalog Number	Dilution
SESN3	Rabbit	Proteintech	11431-2-AP	1:5,000
PARP	Rabbit	abcam	ab191217	1:5,000
Caspase 9	Mouse	Cell signaling	9508	1:5,000
Bcl-XL	Rabbit	Cell signaling	2764	1:2,000
TGF-β	Rabbit	Cell signaling	3711	1:5,000
Fibronectin	Rabbit	Proteintech	15613-1-AP	1:5,000
SERCA2a	Rabbit	21st Century Biochemicals	Custom made	1:5,000
cTnT	Mouse	Novus	MAB18742	1:5,000
GFP	Rabbit	Santa Cruze	SC-8334	1:5,000
GAPDH	Rabbit	Cell signaling	2118	1:5,000
Goat Anti-Mouse HRP	Mouse	Jackson ImmunoResearch	115-035-003	1:10,000
Goat Anti-Rabbit HRP	Rabbit	Jackson ImmunoResearch	111-035-003	1:10,000

Supplementary Table S3. Quantitative RT-PCR primer information

Gene		Sequence (5'to 3')
pri-miR-25	F	5'-CTC ACA GGA CAG CTG AAC ACC- 3'
	R	5'-CCC CCA CAT CTG CAG TGT TG- 3'
pre-miR-25	F	5'-CAG TGT TGA GAG GCG GAG ACT- 3'
	R	5'-GCA CTG TCA GAC CGA GAC AAG- 3'
miR-25-3p	F	5'-CAT TGC ACT TGT CTC GGT CTG A- 3'
SESN 1	F	5'-GCA TGT TCC AAC ATT TCG TG- 3'
	R	5'-TCC CAC ATC TGG ATA AAG GC- 3'
SESN 2	F	5'-GAC CAT GGC TAC TCG CTG AT- 3'
	R	5'-GCT GCC TGG AAC TTC TCA TC- 3'
SESN 3	F	5'-CCA AGC AAA TAC GGC GGA TG- 3'
	R	5'-TGT AGA ACT GGC TCC GCA AG- 3'
Collagen I	F	5'-GCC AAG AAG ACA TCC CTG AAG-3'
	R	5'-TGT GGC AGA TAC AGA TCA AGC-3'
Collagen III	F	5'-ACA GCA GTC CAA TGT AGA TG-3'
	R	5'-GAG CAG GTG TAG AAG GCT G-3'
18S	F	5'-TAA CGA ACG AGA CTC TGG CAT- 3'
	R	5'-CGG ACA TCT AAG GGC ATC ACAG-3'
U6	F	5'-CGC TTC ACG AAT TTG CGT GTC AT-3'
	R	5'-GCT TCG GCA GCA CAT ATA CTA AAA T-3'

Supplementary Figure S1. SESN3 expression following Pre miR-25 transfection in H9c2 cells

H9c2 cardiac myoblast cells were either transfected with Scrambled control or Pre miR-25 for 24 hours. Cells were then fixed, permeabilized, and stained for SESN3 (red) and nuclei (DAPI, blue).

A scale bar indicates 25μm.

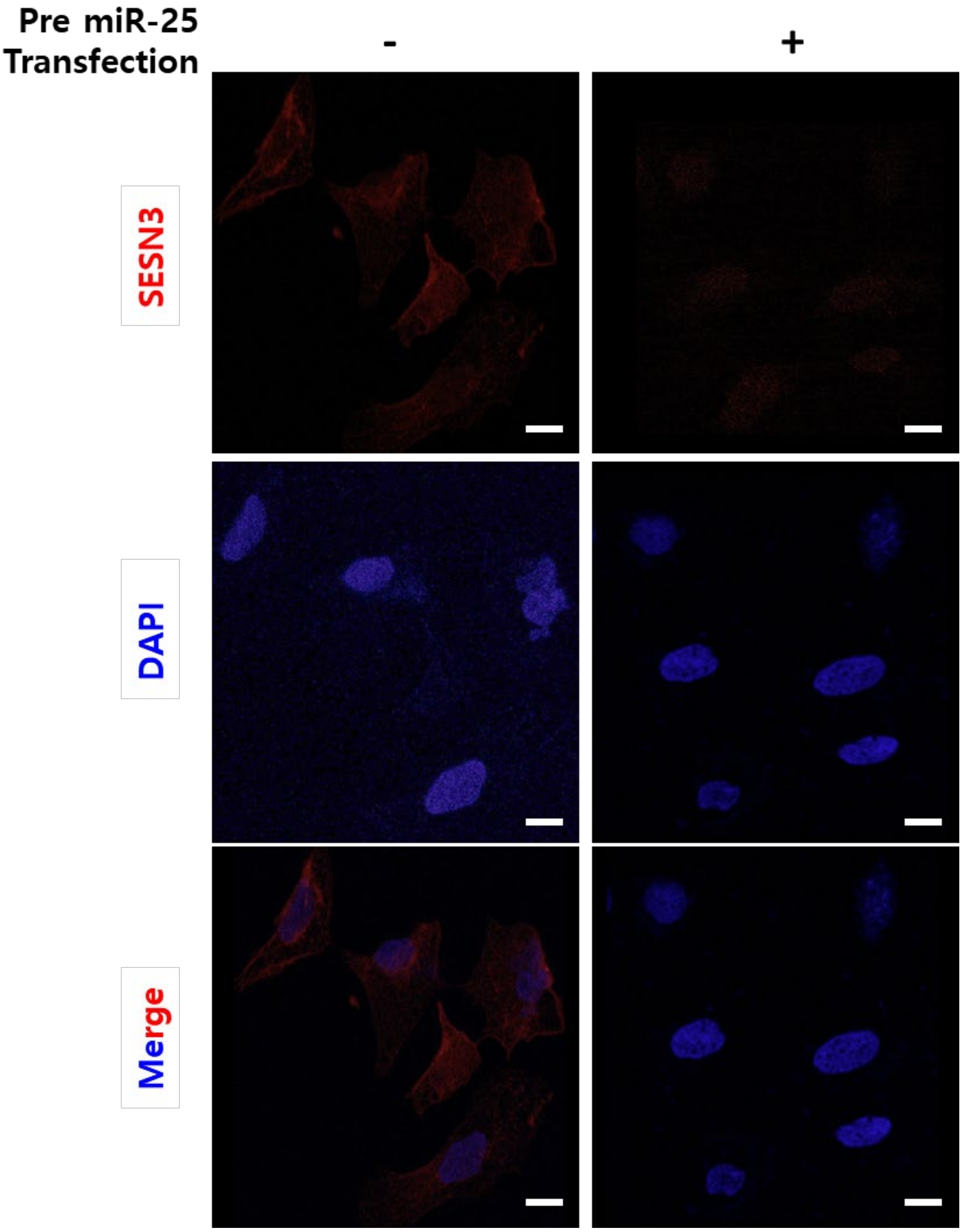

Supplementary Figure S2. Biodistribution of AAV9-EGFP in Various Organs

Two weeks after tail vein injection of AAV9-EGFP, five major organs (Brain, Heart, Lung, Liver and Kidney) were collected from mice receiving a single intravenous AAV9-EGFP administration. These tissues were subsequently subjected to Western blot analysis for GFP expression.

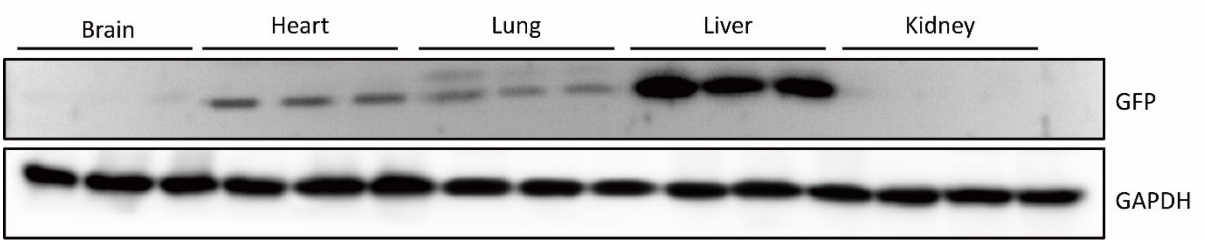

Supplementary Figure S3. miR-25 Expression Levels at 1 Week and 2 Weeks Post-Injury

Quantitative analysis of miR-25 expression in the heart following ischemia/reperfusion (I/R) injury. Mice were administered AAV9-miR-25 TuD via tail vein injection prior to I/R injury. Cardiac tissue was collected at 1 week and 2 weeks post-injury. miR-25 levels were assessed by qRT-PCR and normalized to U6 . Data are presented as mean ± SEM (n = 3 per group). (*** $p < 0.001$ vs Sham, ns: not significant.)

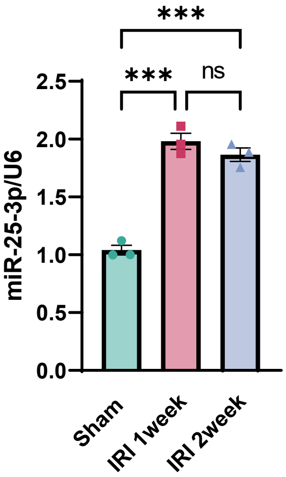

Supplementary Figure S4. Expressions of Collagen Type I and Type III in H9c2 cells under Normoxia and hypoxia/reoxygenation (H/R) conditions following miR-25 TuD treatment

H9c2 cardiac myoblast cells were subjected to normoxia, hypoxia/reoxygenation (H/R), and H/R with miR-25 TuD pretreatment. Collagen Type I and Type III expression levels were assessed by qRT-PCR and normalized to GAPDH. (* $p < 0.05$, ** $p < 0.01$, *** $p < 0.001$ vs Normoxia, # $p < 0.05$ vs H/R, ns: not significant.)

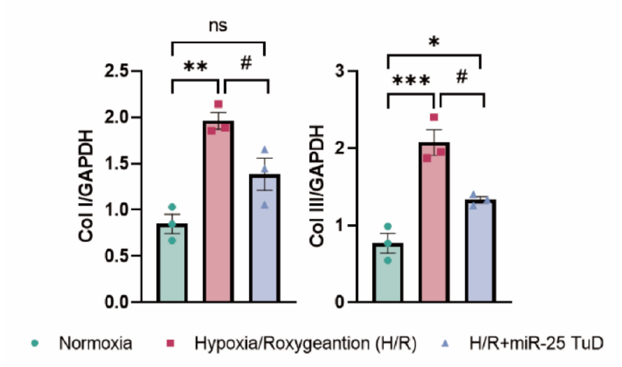

Supplementary Figure S5. Expressions of Collagen Type I and Type III in Cardiac Tissues Following AAV9-miR-25 TuD delivery

Mice were subjected to sham surgery, ischemia/reperfusion (I/R) injury, and I/R with AAV9 miR-25 TuD pretreatment. The AAV9 miR-25 TuD was administered via tail vein injection prior to I/R injury. Collagen Type I and Type III expression levels were assessed by qRT-PCR and normalized to GAPDH.
(** $p < 0.01$, *** $p < 0.001$ vs Sham, # $p < 0.05$, ## $p < 0.01$ vs IRI+AAV9-EGFP, ns: not significant.)

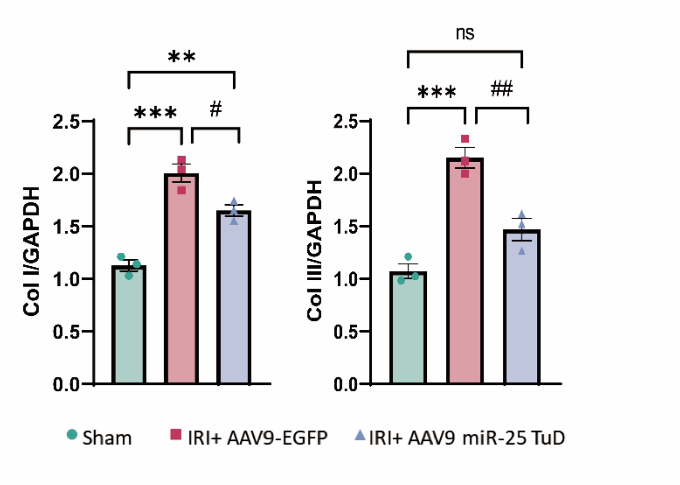

Supplementary Figure S6 The mRNA expressions of SESN1 and SESN2 in H9c2 cells under Normoxia and hypoxia/reoxygenation (H/R) conditions

SESN1 and 2 mRNA expressions were measured in H9c2 cells subjected to various conditions using SESN3 overexpression, miR-25 TuD treatments, and SESN3 knockdown under normoxia and hypoxia/reoxygenation (H/R) conditions. (* $p < 0.05$, *** $p < 0.001$ vs Normoxia, # $p < 0.05$, ## $p < 0.01$, ### $p < 0.001$ vs H/R).

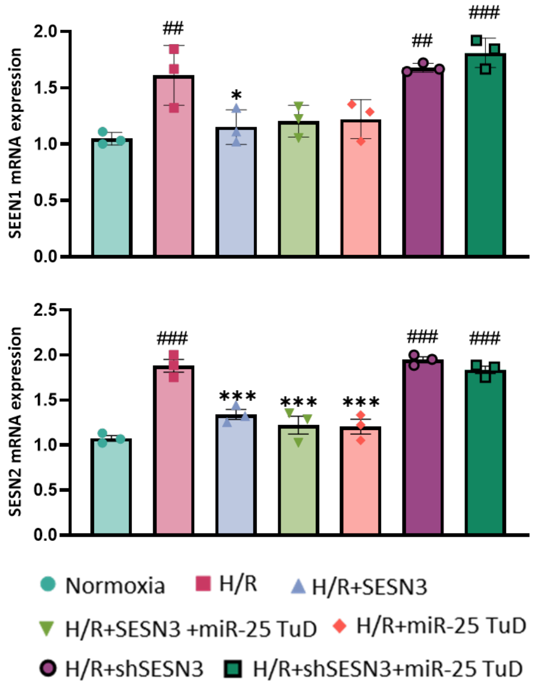

Supplement: Supplementary file 1 [file antioxidants-14-00061-s001.zip › antioxidants-3395516-supplementary.pdf]
